# Trueness and precision of intraoral scanners in the maxillary dental arch: an *in vivo* analysis

**DOI:** 10.1038/s41598-020-58075-7

**Published:** 2020-01-24

**Authors:** Jonas Winkler, Nikolaos Gkantidis

**Affiliations:** Department of Orthodontics and Dentofacial Orthopedics, University of Bern, Freiburgstrasse 7, CH-3010 Bern, Switzerland

**Keywords:** Oral anatomy, Dental equipment, Medical research

## Abstract

Intraoral three-dimensional imaging has gained great interest in dentistry as a mean to generate risk-free imprints of the oral cavity. Accurate intraoral models facilitate proper diagnosis, growth assessment, outcome evaluation, and 3D printing applications. Here, in an actual clinical setup on 12 subjects, we evaluate the trueness and precision of two widely used intraoral scanners (TRIOS 3, 3Shape and CS 3600, Carestream), using an industrial scanner (Artec Space Spider) as a reference. Surface based matching was implemented using the iterative closest point algorithm (ICP). Trueness of the intraoral scans was analyzed by measuring their distance from the reference scan, in the upper buccal front area. Precision was tested through the distance of repeated scans regarding the whole dental arch, following superimpositions in the buccal front and in the whole dental arch area. TRIOS 3 displayed slightly higher precision (approximately 10 μm) compared to CS 3600, only after superimposition on the whole dental arch (p < 0.05). Both intraoral scanners showed good performance and comparable trueness (median: 0.0154 mm; p> 0.05). However, in individual cases and in various, not spatially defined areas, higher imprecision was evident. Thus, the intraoral scanners’ appropriateness for highly demanding, spatially extended clinical applications remains questionable.

## Introduction

Digital three-dimensional imaging has gained great interest in dentistry as a mean to generate an imprint of the oral cavity. Digital dental models can overcome certain drawbacks associated with plaster models, such as patient discomfort and vulnerability. Being also advantageous in terms of cost, time, and space required, digital models will probably soon become the new standard in clinical practice^1^.

Currently there are two ways to generate a digital 3D model: direct intraoral digital impression with an intraoral scanner and extraoral scanning of conventional plaster casts or impressions. In contemporary clinical practice, alginate impressions are still commonly used due to simplicity reasons, adequate accuracy for diagnosis and low costs. However, the intraoral scans are very rapidly incorporated in everyday practice.

Several studies have tested the performance of intraoral scanners both *in vivo* and *in vitro* and concluded that relatively precise 3D dental model representations of a patient’s mouth can be performed^2–4^. The accuracy of intraoral digital scans has also been reported to be clinically adequate as assessed through 2D linear measurements^5,6^ or 3D surface assessments^6–8^. Thus, the models obtained through intraoral scans are satisfactory for diagnostic reasons.

However, direct intraoral scans have been shown to have a degree of imprecision and inaccuracy, attributed to the 3D model generation process^3,7,9^. So far, there are few *in vivo* studies that evaluated complete-arch scans acquired directly in the patient’s mouth. To our knowledge, there is only one study which tested the accuracy of full arch digital impression procedure in a clinical setup, as compared to a gold standard^3^. However, the setup of this study was not fully representative of actual clinical conditions, since the researchers used artificial bodies bonded to the buccal surfaces of teeth as superimposition references, arguing that the scans were optimized in these areas. Furthermore, this study assessed only the buccal tooth surfaces.

Accurate intraoral 3D models facilitate proper diagnosis, growth assessment, and treatment outcome evaluation. Especially for the latter two cases, superimposition of 2D or 3D radiographs is a standard procedure to evaluate morphological changes^10^. The superimposition of 3D digital dental models was suggested to assess changes in the oral cavity in a 3D manner and with high accuracy, avoiding the harming effects of ionizing radiation^11,12^. However, accurate 3D models are a prerequisite to obtain valid results through these promising new tools^13^. Additionally, accurate 3D models are required for the proper fitting of 3D printing applications *in vivo*, which are increasingly incorporated in various dental fields^14^.

Therefore, the aim of this study was to evaluate the efficacy of different intraoral scanners, in terms of trueness and precision, on the representation of the original morphology of the whole dental arch. For this purpose, we assessed whole maxillary arch 3D digital models obtained from direct intraoral scanning with two different scanners, fully representing actual clinical conditions. A high accuracy extraoral scanner was used to provide the gold standard surface models.

## Materials and Methods

Ethical approval was obtained prior to the study by the Swiss Ethics Committee (ID 2017–01659). The methods were carried out in accordance with the relevant guidelines and regulations. All participants signed an informed consent prior to their enrollment in the study.

### Sample

The study sample consisted of 12 (8 M, 4 F) adult volunteers (27–52 years old) who fulfilled the following eligibility criteria:

- no extremes of palatal shape, no visible edema in the attached or removable alveolar bone and on palatal mucosa (visual inspection)
- no extreme malocclusions, no crossbites, no large asymmetries (visual inspection)
- no missing teeth from 2^nd^ molar to 2^nd^ molar
- Caucasian participants
- Before or >2 years after the end of any previous orthodontic treatment

Sample size was determined for the precision outcome based on existing data^8^. We were interested on an effect size of 20 μm. For a power of 90%, an alpha of 0.05 and an SD of 8 μm the effect size having a sample size of 5 is 19 μm. However, since the previous study^8^ is not identical to ours we decided to increased our sample to 10. To account for potential dropouts, we initially enrolled 13 individuals in the study and we finally analyzed 12. The 3D models of one participant were excluded due to double contour in the incisal surfaces.

### Scanners

In total three different scanners were used. These were the CS 3600 (Carestream, Atlanta USA, Software CS Imaging Version 7.0.23.0.d2), the TRIOS 3 (3Shape, Copenhagen, Denmark, Software Version 1.4.7.5), and the industrial scanner Artec Space Spider (Artec3D, Luxembourg, Software ArtecStudio 12 Professional Version 12.1.6.16), respectively.

### Data acquisition

The following data acquisition sequence was followed in all subjects:

1. 3D scan with industrial scanner Artec Space Spider (one time)
2. 3D scans with CS 3600 (two times)
3. 3D scans with TRIOS 3 (two times)
4. 3D scan with CS 3600 (one time)

All scans were obtained by the first author who had more than two years of experience with regular clinical use of intraoral scanners. The same investigator performed all the steps of data generation following relevant training and under close supervision by the senior author.

The first scan was obtained through an industrial handheld scanner (Artec Space Spider) with patients seated on a dental chair in an upright position. The scanner was preheated and calibrated according to manufacturer’s instructions. This scanner was used to scan the buccal side of the subjects’ upper front teeth. Prior to scanning a cheek and lip retractor (Spandex, Hager, Germany) was placed, teeth were dried and a dental coating spray from a pressurized canister was applied according to manufacturer’s instructions (Scansprayplus, Dentaco, Germany). The scan was always started in the middle of the upper jaw and moved laterally and posteriorly. Due to anatomical and physical limitations that did not allow simultaneous access of all Artec Space Spider scanner cameras to the whole dentition it was not possible to properly scan the region posterior to the first premolars in most participants and the palatal aspects of teeth. For this reason, only the buccal anterior surfaces were considered for use in the study.

After detaching the retractor, the participants were asked to brush carefully their teeth with a super soft dental brush (S27, Paro, Switzerland). Coating leftovers were removed by the investigator using water spray.

After a ten-minute rest-period participants were seated again on the dental chair in a horizontal position. Following proper tooth drying, five intraoral scans of the upper jaw were obtained; two scans with CS 3600, two scans with TRIOS 3, and a third scan with CS 3600. The scans were generated according to the manufacturer’s guidelines. Scanning of the maxilla started with the second molar in the first quadrant and ended at the second molar in the second quadrant. Scanning of palatal soft tissues started with the palatal side of the central incisors and moved distally back to the level of the distal end of the second molars. Before completing the whole scan, missed areas were rescanned. Unbroken and smooth digital images were considered scans of acceptable quality to be included in the study.

### 3D data processing

In each sequence of the industrial scanner (Artec Space Spider) all non-essential data, such as irrelevant soft tissues or opposing dentition, were removed using Artec Studio 12 Professional (Version 12.1.6.16, Artec3D) software. Thereafter, the cropped 3D model was exported as an STL file.

3D surface models from all intraoral scanners were also exported as STL files using specific software (CS 3600: CS Imaging, Version 7.0.23.0.d2; TRIOS 3: Trios, Version 1.4.7.5).

The STL-files from Artec Space Spider, CS 3600, and TRIOS 3 were imported into Viewbox 4 software (Version 4.1.0.1 BETA 64, dHAL Software, Kifissia, Greece). There, the 3D surface models were manually cropped within 1 mm from the sulcus to include only tooth surfaces. The final 3D model included the first six teeth of each quadrant. From the Artec Space Spider derived models, only the buccal surfaces of the upper anterior teeth were included, since they were considered valid for reasons described previously. Following this process, the subsequent 3D models were exported and saved again as STL files that comprised the final 3D models analyzed in the study. Each of these models consisted of approximately the following number of vertices: 50000 for the Artec Space Spider, 60000 for CS 3600, and 60000 for TRIOS 3 derived models, respectively.

### Trueness and precision testing

The final 3D models were superimposed in Viewbox 4 using the software’s implementation of the iterative closest point algorithm (ICP)^15^ with the following settings: 100% estimated overlap of meshes, matching point to plane, exact nearest neighbor search, 100% point sampling, and 50 iterations.

Trueness of each test group (CS 3600 and TRIOS 3) was assessed in the front segment and more specifically in the buccal surfaces of the upper anterior teeth, since this was the only area that was adequately captured by the industrial scanner. For this purpose, each STL file from each intraoral scanner was superimposed with the reference scan from the Artec Space Spider scanner. The Artec Space Spider scan served as gold standard reference, since this scanner shows accuracy and precision beyond what is achievable with intraoral scanner systems. The superimposition reference area included the buccal surface of the maxillary incisors and the mesiobuccal surfaces of the upper canines. The incisal edges and the sulcus were cropped to a distance of 1–2 mm. Trueness was calculated by measuring the MAD (Mean Absolute Distance) of each model from the high accuracy industrial scanner model following a best-fit registration. Zero MAD would imply maximum trueness, whereas increasing MAD would mean decreasing trueness of the model. Respective colour maps are presented for all cases to allow the qualitative assessment of the outcome and the localization of potential differences.

Precision was assessed through superimposition of 3D models generated from repeated CS 3600 and TRIOS 3 scans, using two different superimposition reference areas. The first included all maxillary teeth from first molar to first molar. The second included only the front buccal teeth area that was used to assess trueness, since trueness was defined only for this area. The MAD distance between the corresponding whole dental arch 3D models, obtained after repeated scan superimpositions on the two reference areas, was the outcome of interest for precision testing. In this case also, respective colour maps are presented.

Precision of the Space Spider scanner was also tested at 3 randomly selected subjects (https://www.random.org/) to verify its appropriateness to be used as a gold standard.

### Statistical analysis

Statistical analysis was carried out by using the SPSS (v.20.0, SPSS Inc., U.S.A) software.

Raw data were tested for normality through the Shapiro-Wilk test and did not have a normal distribution in certain cases. Thus, non-parametric statistics were applied.

Differences in the measured variables were tested in a paired manner through the Friedman test. In case of significant outcomes, pairwise comparisons were performed through the Wilcoxon-signed rank test.

In all cases, a two-sided significance test was carried out at an alpha level of 0.05. The level of significance was adjusted according to the Bonferroni correction in case of pairwise a posteriori multiple comparison tests, to reduce the possibility for false positive results.

## Results

### Trueness of intraoral scanners

**Figure 1:**
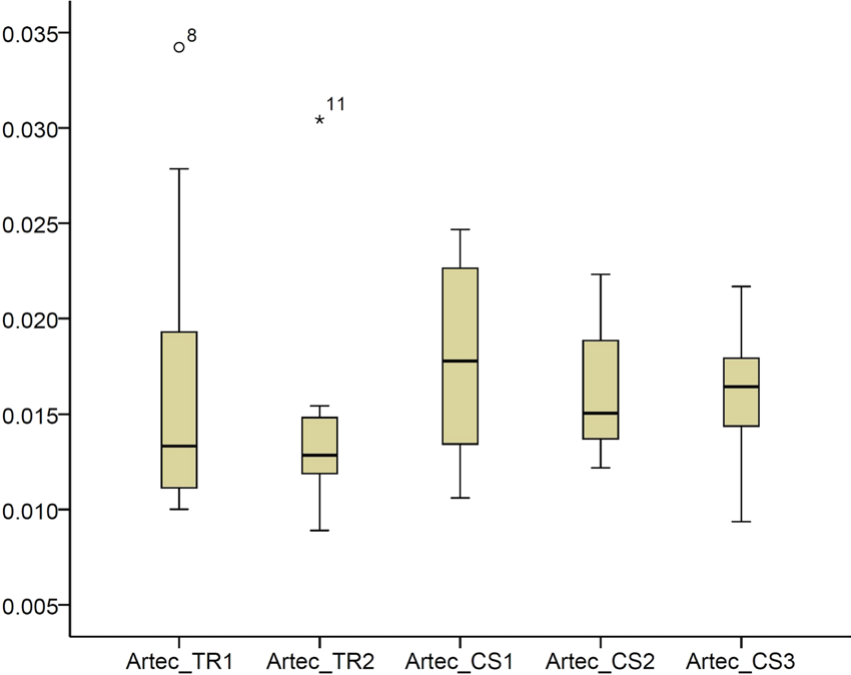
Box plots showing the trueness of the repeated intraoral scans with two different scanners in millimeters (n = 12, p = 0.176, Friedman test). The upper limit of the black line represents the maximum value, the lower limit the minimum value, the box the interquartile range, and the horizontal line the median value. Outliers are shown as black dots or stars, in more extreme cases. CS1, CS2, CS3: CS3600 repeated scans. TR1, TR2: TRIOS3 repeated scans. Artec: Artec Space Spider scan.

**Figure 2:**
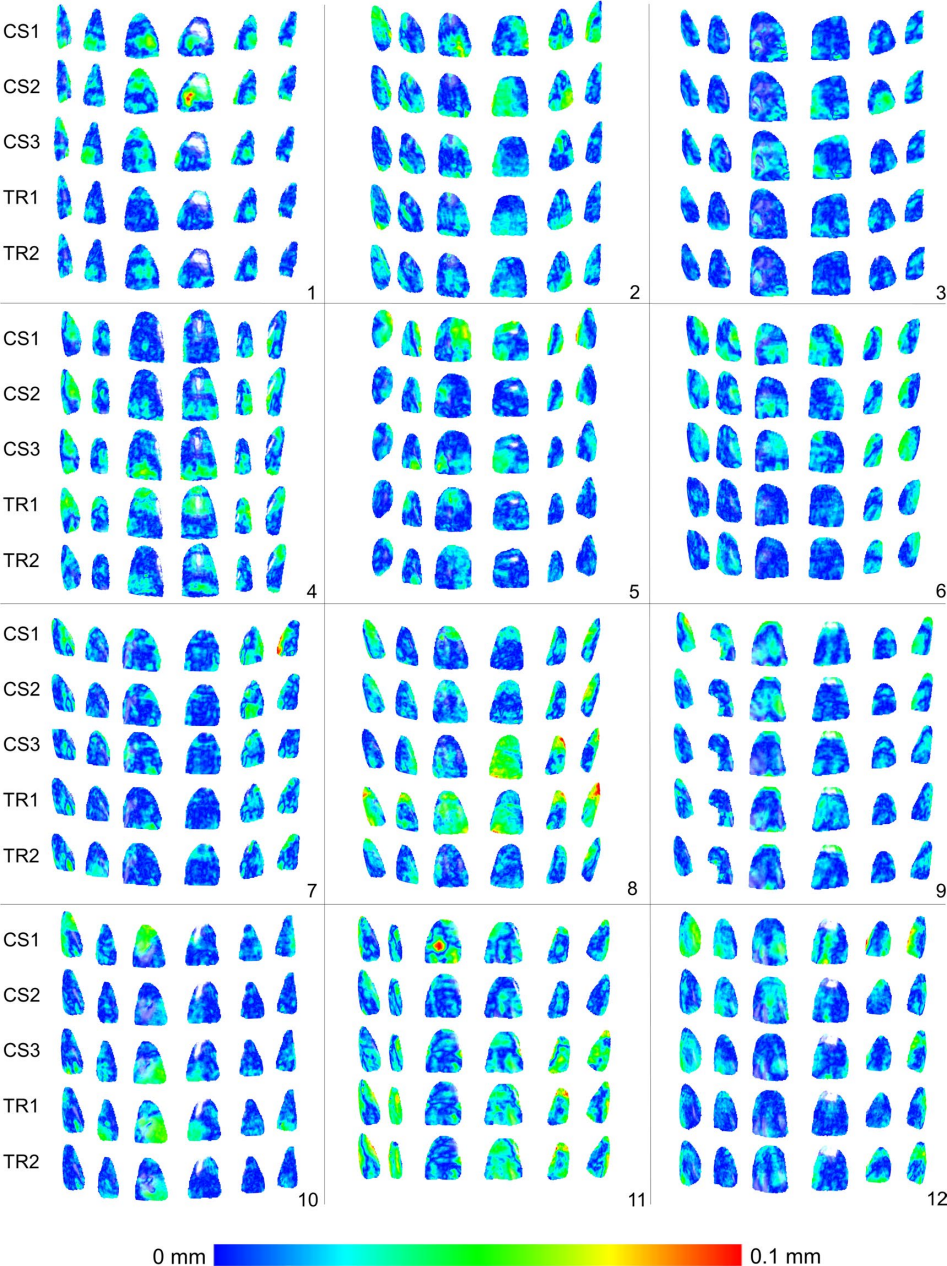
Colour maps showing the trueness assessment measured through the MAD of the CS3600 and TRIOS3 intraoral scanners from the gold standard scans, in the upper buccal front teeth area. Three scans obtained through CS3600 (CS1, CS2, CS3) and two scans through TRIOS3 (TR1, TR2) were assessed (n = 12 for each set of scans).

The median trueness of both intraoral scanners in the buccal regions of the upper anterior teeth, assessed as the MAD of the intraoral models to that obtained through a high accuracy extraoral scanner was 0.0154 mm (range: 0.0089, 0.0771; Fig. 1). There was no difference between the trueness of repeated scans or of different scanners (CS1/CS2/CS3 median: 0.0164 mm, range: 0.0094, 0.0247; TR1/TR2 median: 0.0129 mm, range: 0.0089, 0.0342) (Friedman test, p = 0.176). Two outliers were present for TRIOS 3 (Fig. 1). The respective colour maps (Fig. 2) showed high trueness in the overall tested area, with no specific inaccuracy or asymmetry patterns detected in the sample.

### Precision of intraoral scanners

The precision of the intraoral scanners (TRIOS 3 and CS 3600) was tested after superimposing the dental arch surface models obtained from repeated scans. The MAD of the whole dental arch area was the testing variable.

**Figure 3:**
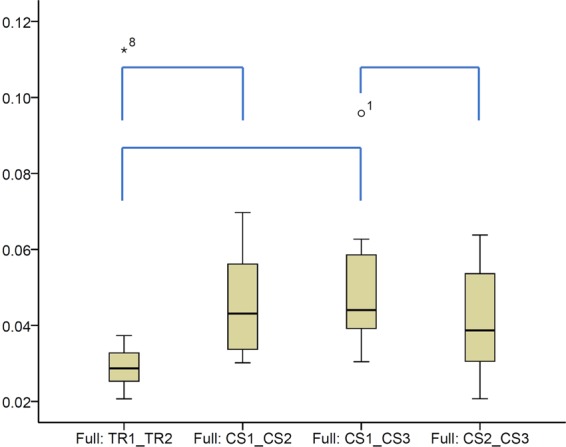
Box plots showing the precision (millimeters) assessment measured through the MAD of the whole dental arch area between repeated scans with the two different scanners (CS3600 and TRIOS3), when the whole dental arch area was used as superimposition reference (Full). The upper limit of the black line represents the maximum value, the lower limit the minimum value, the box the interquartile range, and the horizontal line the median value. Outliers are shown as black dots or stars, in more extreme cases. Lines connecting pairs of box plots imply significant differences between them (Wilcoxon signed rank test, p < 0.01). CS1, CS2, CS3: CS3600 repeated scans. TR1, TR2: TRIOS3 repeated scans.

**Figure 4:**
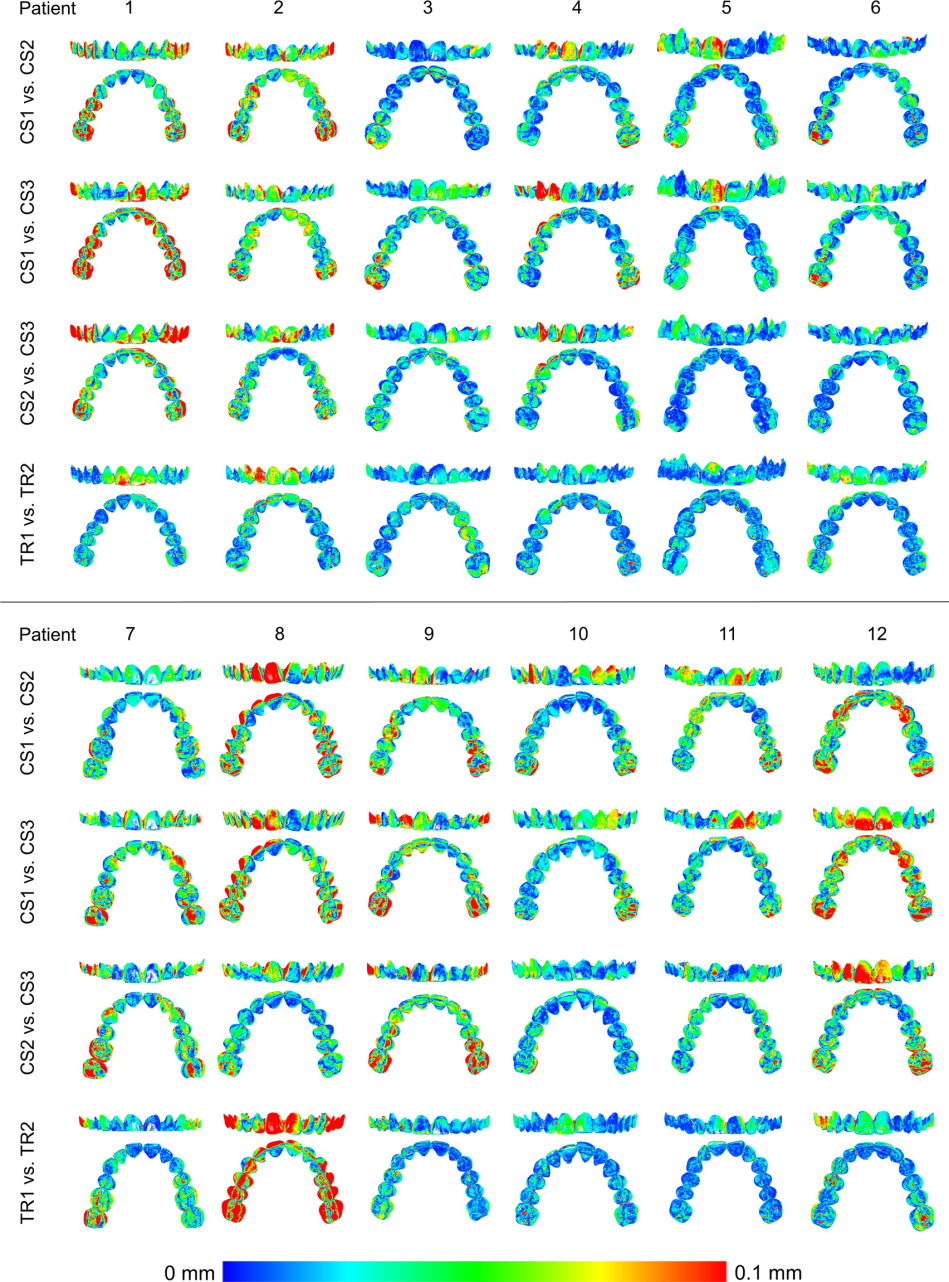
Colour maps showing the precision of the intraoral scanners. Three scans form CS3600 (CS1, CS2, CS3) and two scans from TRIOS3 (TR1, TR2) were assessed. The region for superimposition was the whole maxillary dental arch.

When superimpositions were performed on the whole dental arch surface, a significant difference was identified between the precision of the two scanners, with the TRIOS 3 showing better precision than the CS 3600 in two out of the three cases tested (Fig. 3, Friedman test, p = 0.003, Wilcoxon-signed rank test, p < 0.05). The overall difference of the median precision measurements between the two scanners was approximately 10 μm (0.0098 mm). However, the lowest precision was detected for TRIOS 3 scans of a specific patient. No specific imprecision patterns were observed in the respective colour maps, since the errors were distributed equally between the different areas of the arch. However, in certain cases the imprecision was higher than in others. There was also significant variation within the arches, with local imprecisions reaching in several cases relatively high values (Fig. 4).

**Figure 5:**
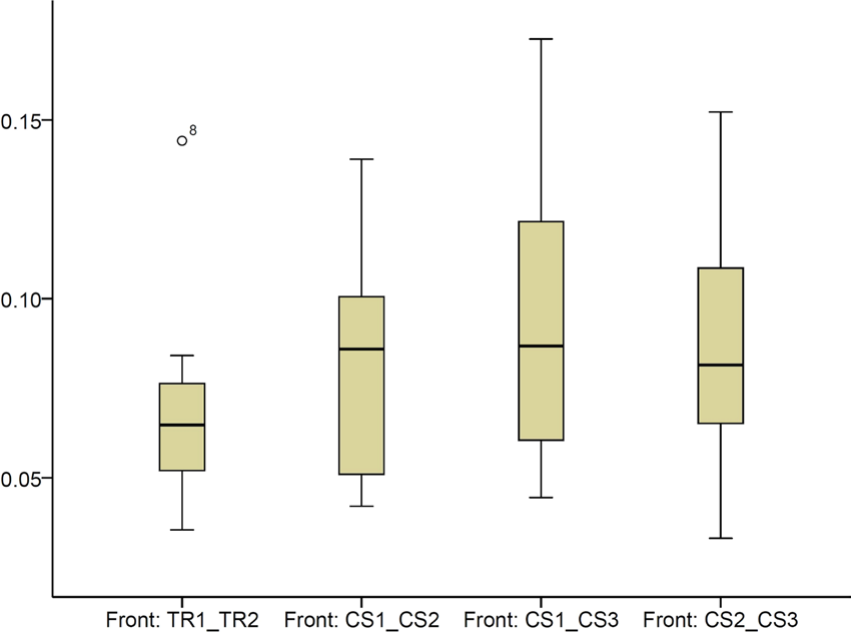
Box plots showing the precision (millimeters) assessment measured through the MAD of the whole dental arch area between repeated scans with the two different scanners, when only the upper buccal front teeth area was used as superimposition reference (Front). The upper limit of the black line represents the maximum value, the lower limit the minimum value, the box the interquartile range, and the horizontal line the median value. Outliers are shown as black dots. CS1, CS2, CS3: CS3600 repeated scans. TR1, TR2: TRIOS3 repeated scans.

**Figure 6:**
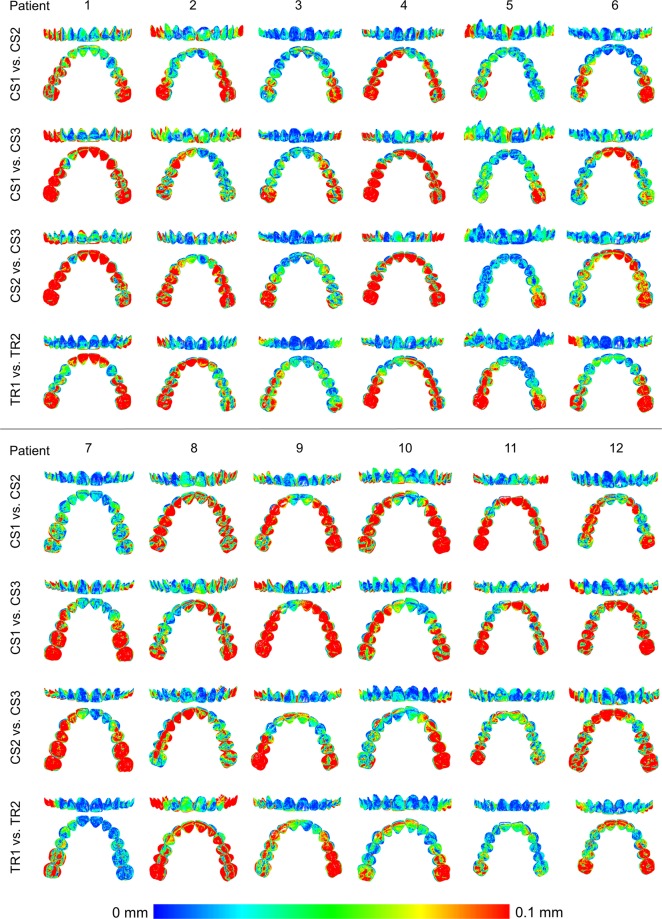
Colour maps showing the precision of the intraoral scanners. Three scans obtained through CS3600 (CS1, CS2, CS3) and two scans through TRIOS3 (TR1, TR2) were assessed. The reference region for superimposition was the upper buccal front teeth area.

When superimpositions were performed on the buccal front teeth area, similar precision of the scanners was evident (Fig. 5, Friedman test, p = 0.296), (TRIOS 3 median: 0.0648, range: 0.0354, 0.1442; CS 3600 median: 0.0860, range: 0.0331, 0.1726). This suggests that in smaller dental arch areas the two scanners perform similarly, whereas considering the whole dental arch, TRIOS 3 shows slightly better precision. The respective colour maps show that the imprecision is evident primarily in the posterior arch areas and in the palatal side of the anterior teeth (Fig. 6). This is an expected finding, since in this case the 3D models were superimposed in the buccal anterior teeth area through the best fit algorithm. Also in this case, individual variation is evident and local imprecision is often relatively high (Fig. 6).

### Precision of the reference scanner

**Figure 7:**
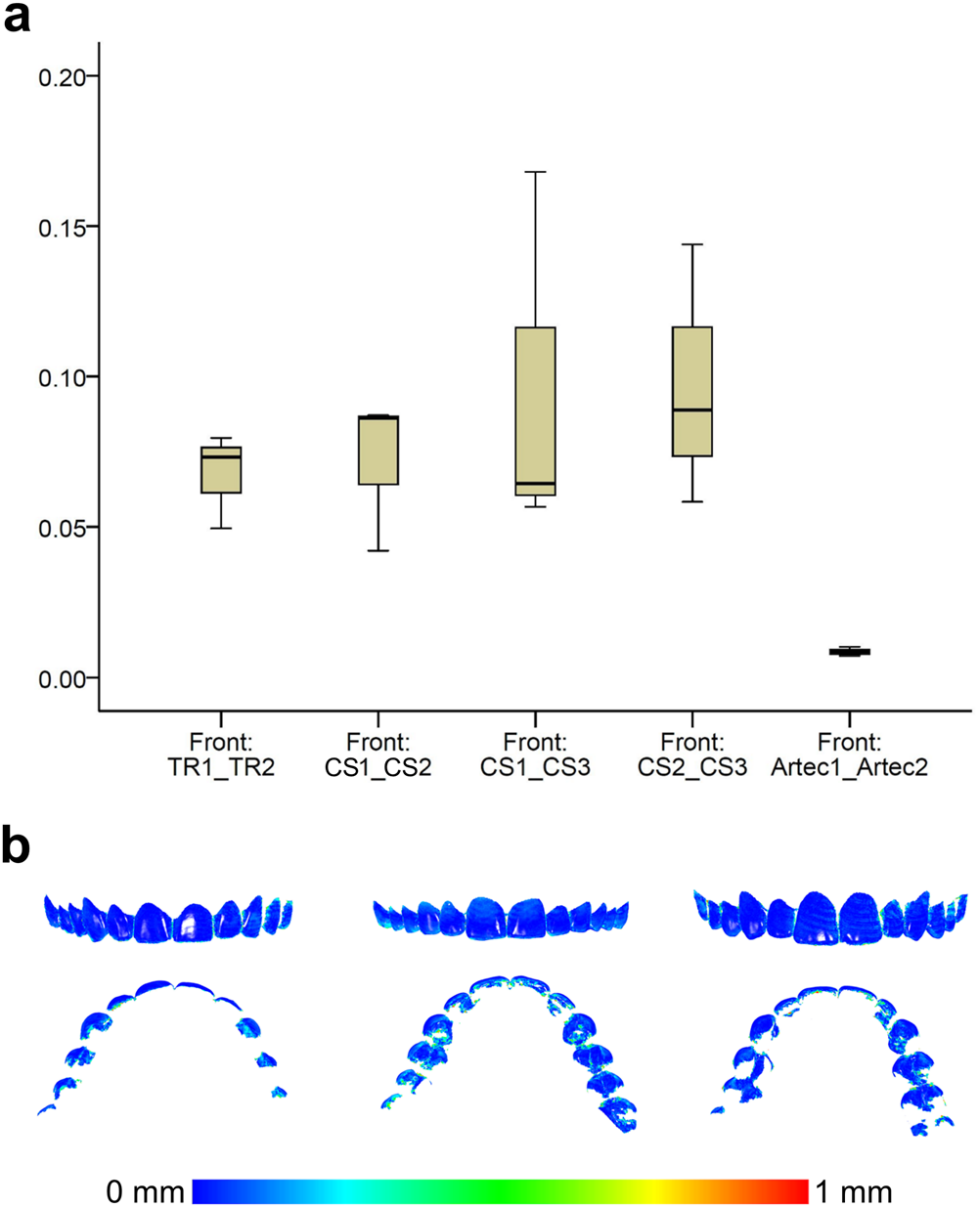
(**a**) Box plots showing the precision (millimeters) assessment of the gold standard scanner versus the intraoral scanners (n = 3), measured through the MAD of the whole dental arch area between repeated scans, when only the upper buccal front teeth area was used as superimposition reference. The upper limit of the black line represents the maximum value, the lower limit the minimum value, the box the interquartile range, and the horizontal line the median value. CS1, CS2, CS3: CS3600 repeated scans. TR1, TR2: TRIOS3 repeated scans. Artec1, Artec2: Artec Space Spider repeated scans. (**b**) Colour maps showing the absolute distances between corresponding points of surface models obtained through repeated scans with the Artec Spice Spider scanner.

The precision of the Artec Spider scanner in the area of interest was excellent and it was much superior to that of the intraoral scanners confirming the choice to use it as a gold standard for the study (Fig. 7). Small imprecision was evident in the interdental spaces or at the incisal edges and the occlusal fissures of the teeth, but these areas were not used in the study.

## Discussion

The aim of this study was to assess trueness and precision of digital intraoral scanners on representation of the original morphology of the whole maxillary dental arch. An industrial high accuracy scanner (Artec Space Spider) was used as gold standard. Both intraoral scanners, TRIOS 3 and CS 3600, showed comparable trueness in the upper buccal front region. Precision was also comparable when the repeated models were superimposed in the upper buccal front region. However, TRIOS 3 showed slightly higher precision, when the whole model was used as superimposition reference. The difference in precision between the two scanners was approximately 10 μm and this can be considered clinically insignificant. Overall, both intraoral scanners showed good performance and comparable trueness and precision values in whole maxillary arch representations. However, in individual cases and in various areas of the arch, higher imprecisions were evident.

The present study is the first to use a method for assessing the trueness of whole upper dental arch intraoral scans in actual clinical conditions. This was made possible by using the upper buccal anterior teeth area as gold standard reference. The basic idea was to use a high accuracy industrial optical scanner (Artec Space Spider) to generate an accurate reference data set, which was then used as gold standard. Indeed, the adequate performance of the reference scanner^16^ was verified here by the excellent precision that it showed in repeated scans.

In the past, certain *in vivo* studies used conventional impressions as references^17^. Other studies measured only the precision through repeated scans^8^. With these approaches, no conclusion about trueness can be drawn. Another study tested the conventional and digital imprint of a configuration of metal reference spheres fixed with composite to teeth, using a standardized application aid^18^. However, this method tested inaccuracies only on the spheres and not in the actual anatomical structures of interest. So far, there is only one study in the literature that used a gold standard reference^3^. For this purpose, a reference model of the buccal surface of upper teeth was generated with a high accuracy industrial scanner. However, superimposition for trueness measurements was done only on four reference bodies that were bonded in the buccal maxillary tooth surfaces, prior to model acquisition. This approach is considered superior to the already existing studies, but it does not fully represent actual clinical conditions. Furthermore, the latter study assessed only the buccal surfaces of the maxillary teeth.

In the present study, we assessed the whole dental arch using an actual clinical setup, with no artificial items on the teeth surfaces. For this purpose, we used the upper buccal anterior teeth surfaces, captured by a high accuracy scanner, as gold standard reference structures. Any potentially inaccurate areas, such as interproximal regions or incisal edges were excluded. A gold standard reference model of the whole dental arch was not possible to be obtained, since the reference scanner is not constructed for intraoral use. Thus, in occlusal, palatal, and posterior areas of the arch it was not possible to obtain valid surface information, since all the sensors of the scanner could not reach the area of interest at the same time. The high performance of this scanner was verified in this study by the very high precision of the Artec Space Spider scanner, which was much superior to that of the intraoral scanners. Thus, this approach may be at the moment an adequate approximation of the truth and therefore a useful tool for such purposes.

Certain limitations are also present when using such a reference system, since this scanner is originally not intended for intraoral applications. First of all, to successfully scan shiny and reflecting objects, such as tooth surfaces, coating has to be applied. The powder has the advantage of more regular light reflection, which could lead to better matching process^8^. On the other hand, great care has to be taken in dispersing the coating in a homogeneous thickness. Some localized minimal artefacts, not visible upon clinical inspection, cannot be ruled out. Therefore, areas with greater uncertainty regarding coating thickness, such as interproximal areas and incisal edges, were cropped in our models. In the present study, the final surface model obtained through this procedure showed excellent reproducibility, verifying the proper application of the method. Furthermore, if a uniform change of the buccal surface area was evident due to the coating, this would have resulted in homogenous distance between the intraoral scans that did not require any coating and the reference scan. This was not the case in any of the tested models, and thus we concluded that the effect of this factor was not significant.

The intraoral scanners tested in the present study are widely used in the dental field and correspond to the highest standards that the currently available intraoral scanners can reach^3,4^. Concerning the trueness of the intraoral scanners, TRIOS 3 and CS3600 showed no statistically significance difference in intra- or inter-system evaluations. This result is supported by visual inspection of the color maps of the individual cases, where equal distribution of deviations with no detectable asymmetry pattern were seen. Previous studies report contradictory findings regarding different scanners that are available in the market. Although not using the same design and intraoral scanners with our study, Nedelcu *et al*.^3^ and Kuhr *et al*.^18^ found higher trueness of TRIOS 3 and 3 M (True definition) compared to Ominicam (Cerec).

Our study showed varying results for the precision of the intraoral scanners, depending on whether the models were superimposed on the buccal anterior teeth area or on the whole upper dental arch. In the first case, the precision measured through MAD of corresponding models varied around 20–180 μm and it was similar for both scanners. When the whole dental arch was used as superimposition reference area, precision varied around 20–70 μm and was significantly higher for TRIOS 3, in two out of three cases tested. However, though the median difference was quite small (approximately 10 μm) and the highest imprecision was detected for a TRIOS 3 scan. Thus, in partial dental arch models the two scanners perform similarly, whereas considering the whole dental arch, TRIOS 3 shows in most cases slightly better precision. Though the differences between scanners were of limited extent, they might have implications both for 3D superimposition outcomes^13^ as well as for 3D printing applications^14^. On the other hand, such small differences might be easily eliminated by the continuous technical advancements of the hardware and software systems used for intraoral imaging.

Differences in the precision of various intraoral scanners were already reported in previous studies. In most cases the precision of older scanning systems was lower compared with newer systems^7^. However, up to date systems show also significant variations^3^. The cause for this is difficult to be determined. Precision could be influenced by several factors, such as the actual measurement sensitivity of the intraoral scanner, the image construction, the software algorithm for post-processing of the 3D-model (3D rendering process), the scanning protocol, and the operator bias. It seems that the precision of complete-arch scans equals^7^ or exceeds^3^ that of certain conventional impression materials, such as irreversible hydrocolloid. This shows the potential of 3D-scans as an equivalent or even better alternative to certain conventional impression methods.

Both the intraoral scanners showed larger standard deviations in precision measurements compared with the high precision industrial scanner. This can be attributed to the image acquisition process or to the post-processing of the data^19^. Although all scans were taken by the same well-trained dentist, who was familiar with all scanning systems, operator bias cannot be excluded.

The respective colour maps following superimposition on the buccal anterior area show that the imprecision is evident primarily at the posterior arch areas and also at the palatal side of the anterior teeth. This is expected when the best fit of the models is performed on the buccal anterior region. Through a different study design, Nedelcu *et al*.^3^ found similar results for TRIOS 3, 3 M True definition, and Cerec Omnicam, with highest precision in the buccal front area, when the arches were superimposed on artificial buccal reference bodies. Unfortunately, limited information is reported in this study regarding precision. However, the colour maps of one subject, which are presented in the study, show precision in the front region around 0–40 μm and in the posterior region around 100 μm or higher.

To avoid the effect of the location of the superimposition area on outcomes, which inevitably shows imprecision in the opposite sides of the model, and obtain more clinically relevant results, a more thorough assessment of the whole dental arch was performed through whole arch superimpositions in all our cases and the respective colour maps were shown. Surprisingly, no specific pattern of error is evident in these images and the differences cannot be localized to one specific area or side of the dental arch. Thus, the existing belief that the scanners might not work well on the posterior areas of the arch was not confirmed and could primarily be related to the selection of the superimposition reference areas that was performed in the previous studies. Following, a best fit superimposition in the anterior part of the arch, it is likely that one will find larger distances between corresponding models in the posterior areas.

A limitation of the present study is that the trueness validation was based only on the upper buccal front teeth. This was the only possibility, since the reference scanner was not intended for intraoral use and it was impossible to capture other areas of the arch with adequate accuracy. Another limitation is that only the maxillary arch was assessed. This strategy was implemented to avoid extreme fatigue of the participants. In any case, we do not expect different performance of the scanner in the opposing dental arch, but this remains to be tested.

## Conclusions

Through a novel approach for trueness assessment in real clinical conditions, the present *in vivo* study demonstrated that the CS 3600 and TRIOS 3 intraoral scanners show good performance and are capable of representing the whole maxillary dental arch, at least for regular clinical use. Both intraoral scanners showed comparable trueness in the upper buccal front region. Precision of the whole upper dental arch was also comparable when the repeated models were superimposed in the upper buccal front region. TRIOS 3 showed slightly higher precision (approximately 10 μm), when the whole model was used as superimposition reference, but it also showed the highest imprecision in a specific case.

However, in individual cases and in various, not spatially defined areas of the arch, higher imprecision was evident. In a small spatial scale, there is considerable variation in scanner performance between and also within cases. Thus, the usage of intraoral scanners in highly demanding clinical applications, considering the whole arch, remains questionable.

## Data Availability

The datasets generated and/or analyzed during the current study are available from the corresponding author on reasonable request.
